# Electronic structure and its external electric field modulation of PbPdO_2_ ultrathin slabs with (002) and (211) preferred orientations

**DOI:** 10.1038/s41598-017-07212-w

**Published:** 2017-07-31

**Authors:** Yanmin Yang, Kehua Zhong, Guigui Xu, Jian-Min Zhang, Zhigao Huang

**Affiliations:** 1 Fujian Provincial Key Laboratory of Quantum Manipulation and New Energy Materials, College of Physics and Energy, Fujian Normal University, Fuzhou, 350117 China; 2 Fujian Provincial Collaborative Innovation Center for Optoelectronic Semiconductors and Efficient Devices, Xiamen, 361005 China

## Abstract

The Electronic structure of PbPdO_2_ with (002) and (211) preferred orientations were investigated using first-principles calculation. The calculated results indicate that, (002) and (211) orientations exhibit different electric field dependence of band-gap and carrier concentration. The small band gap and more sensitive electric field modulation of band gap were found in (002) orientation. Moreover, the electric field modulation of the resistivity up to 3–4 orders of magnitude is also observed in (002) slab, which reveals that origin of colossal electroresistance. Lastly, electric field modulation of band gap is well explained. This work should be significant for repeating the colossal electroresistance.

## Introduction

In recent years, spin-gapless semiconductors(SGS)^1–4^ with unique properties of semiconductors and half metals simultaneously, analogous to the so called “half semimetal” by Liu *et al*.^5^, have attracted extensive attention due to their potential application in spintronic devices. In comparison with other half semimetals such as toxic Hg-based IV–VI compounds, uninjurious PbPdO_2_-based half semimetals seemed to be more compatible to oxide spintronics devices because they were very sensitive to external pressure, magnetic field and electric field as well. Since PbPdO_2_ half semimetal was predicted by local density approximation (LDA) calculations^2^, magnetic and electric behaviors of PbPdO_2_-based semiconductors had been extensively investigated both theoretically and experimentally^6–12^. Wang *et al*.^6^ reported the colossal electroresistance of PbPd_0.75_Co_0.25_O_2_ film, whose resistivity decreased as the operating current increased. Lee *et al*.^7^ reported the positive magnetoresistance of PbPd_0.9_Cu_0.1_O_2_ and negative magnetoresistance of PbPd_0.9_Zn_0.1_O_2_ samples, which might be attributed to the local structural disorder such as Pd/O deficiencies. Tang *et al*.^8^ explained the observed coexistence of the ferromagnetism, paramagnetism and antiferromagnetism in Co-doped PbPdO_2_ film using a carrier-mediated bound magnetic polaron (BMP) model. Chen *et al*.^9^ found that Pd deficiency in PbPdO_2_ would cause increased O 2p-Pb 6p and decreased O 2p-Pd 4d hybridizations, which induces a small band gap and hence reducing conductivity. Srivastava *et al*.^10^ reported theoretically that the couplings between Co spins were mediated through the Pd and O atoms rather than Pb atoms. Moreover, the colossal electroresistance should be associated with the external electric field modulation of the band gap for PbPdO_2_. To agree with this view, the electric field modulation of the band gap in two-dimensional materials had recently been studied^13–17^. Zheng *et al*.^13^ reported that applied electric field along different direction would weaken or enhance the equivalent field in zigzag BN nanoribbons, resulting in the band gap decrease or increase due to the Stark effect. Liu *et al*.^15^ found that MoS_2_ displayed different band gap modulation for different conformations of MoS_2_ bilayer due to the Stark effect. However, up to now, there was no convincing explanation for different magnetic behaviors and electronic transportation in PbPdO_2_-based semiconductors. Especially, the colossal electroresistance of PbPdO_2_–based semiconductors had hardly been repeated experimentally well, which was worth studying in theory.

In this paper, based on the fact that PbPdO_2_ films with (002) and (211) preferred orientations have about 0.03 eV and 0.20 eV^18^, (002) and (211) orientation slabs of PbPdO_2_ were selected and the corresponding band-gap modulations under electric field were investigated using first principle calculation. It was found that band-gap modulations of (002) orientation was different from that of (211) orientation. The calculated results could be consistent with the reported experimental measurements. At last, the charge polarization theory was applied to address the potential mechanism of electronic behaviors in PbPdO_2_–based semiconductors.

## Results

**Figure 1:**
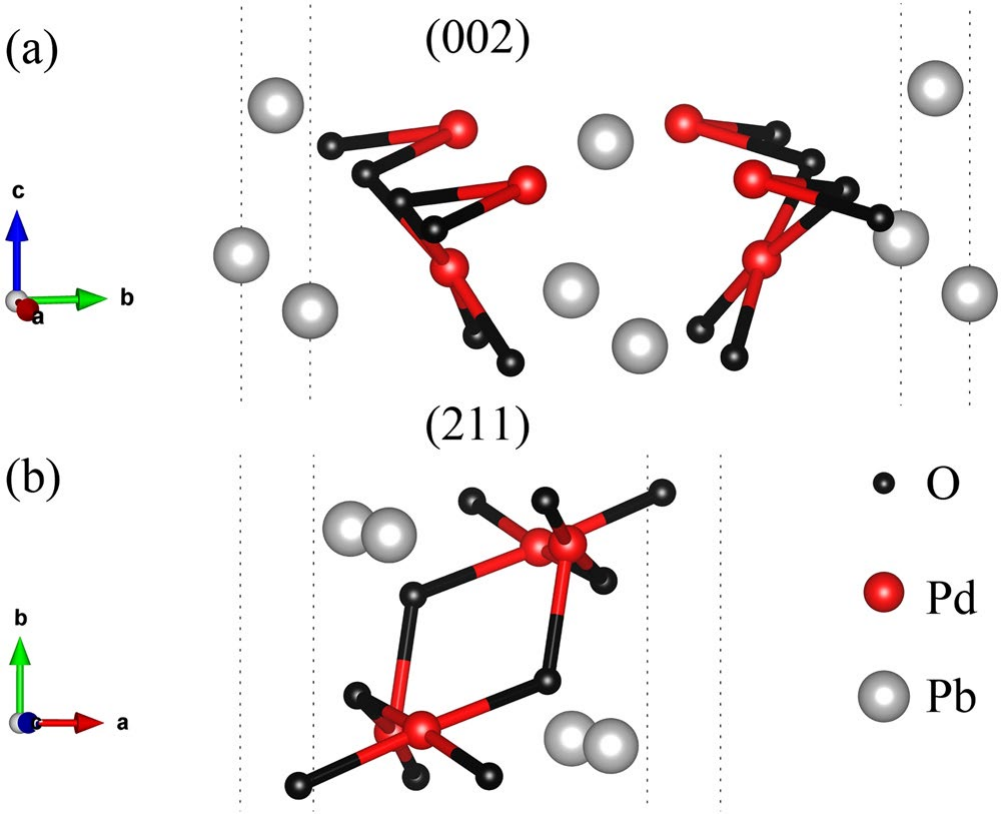
Side view of atomic structures of PbPdO_2_ ultrathin films with (**a**) (002) orientation and (**b**) (211) orientation. Red, gray and black spheres represent Pd, Pb and O, respectively.

Figure 1 shows the slab structures of (002) and (211) orientation in PbPdO_2_. In our work, the thickness of (002) and (211) slab is 3.57 Å and 4.22 Å, respectively. There are four possible terminations of (002) orientation slab, but they have the same value of band gap. As shown in Fig. 1, the (002) orientation slab displays an asymmetric configuration consisting of 8 atoms (along c-axis) and a Pd-O layer with a square-planar coordination, which maintains the vital structure properties of PbPdO_2_. The (211) orientation slab presents a symmetric configuration of the center inversion consisting of 12 atoms (along b-axis). In the case of (211) orientation slab, there is only one termination on the surface due to its inversion symmetry along b-axis direction.

**Figure 2:**
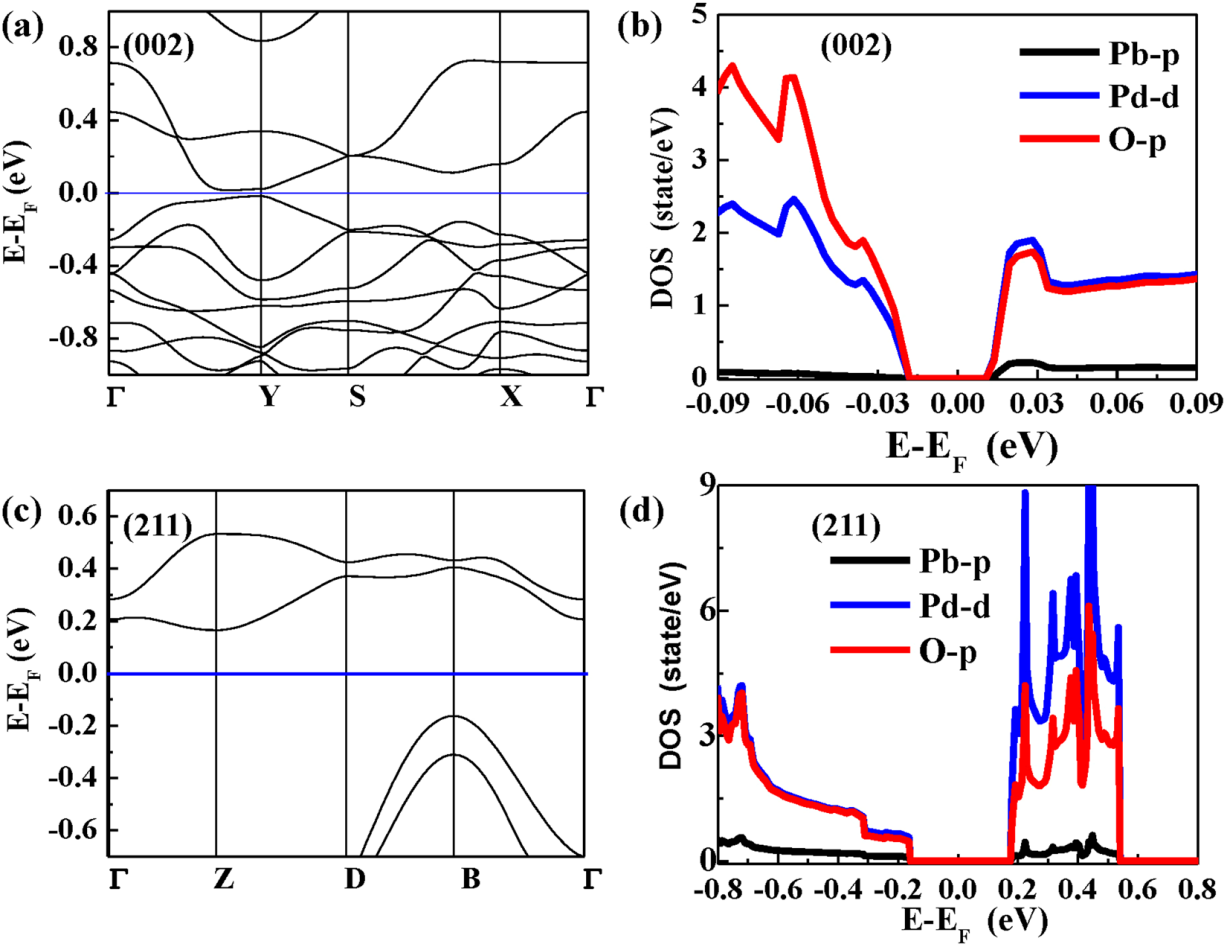
(**a**) Band structures and (**b**) orbital-resolved partial DOS of (002) orientation slab; (**c**) Band structures and (**d**) orbital-resolved partial DOS (211) orientation slab.

Figure 2(a)–(d) show band structures and site-decomposed densities of states of Pb, Pd, and O for (002) and (211) orientation slabs, respectively. It was clear from Fig. 2(a,c) that (002) orientation slab presents a small band gap of 0.03 eV slightly diverging the Y point, which was much smaller than that 0.3 eV of (211) orientation slab. The calculated values of (002) and (211) orientation slabs are consistent with our experimental those with 0.03 and 0.20 eV^18^. DOSs from Fig. 2(b,d) reveal that the density of electronic states of (002) and (211) orientation slabs at CBM (conduction band minimum) and VBM (valence band maximum) were dominated by Pd-4d and O-2p states, which is consistent with previous reported results^7, 9^. Furthermore, different contribution of Pd-4d and O-2p states below and above Fermi energy level (*E*_F_) are also observed in (002) and (211) orientation slabs. In the case of (002) orientation slab, O-2p states contribution is larger than that of Pd-4d states below Fermi energy level while there is no distinct difference between O-2p and Pd-4d states above Fermi energy level. In contrast, (211) orientation slab exhibits the opposite behavior of O-2p and Pd-4d states above Fermi energy level. Thus, it is reasonably expected that Pd-O bonding in the configuration would play an important role in peculiar electric and magnetic performances in PbPdO_2_-base semiconductors.

**Figure 3:**
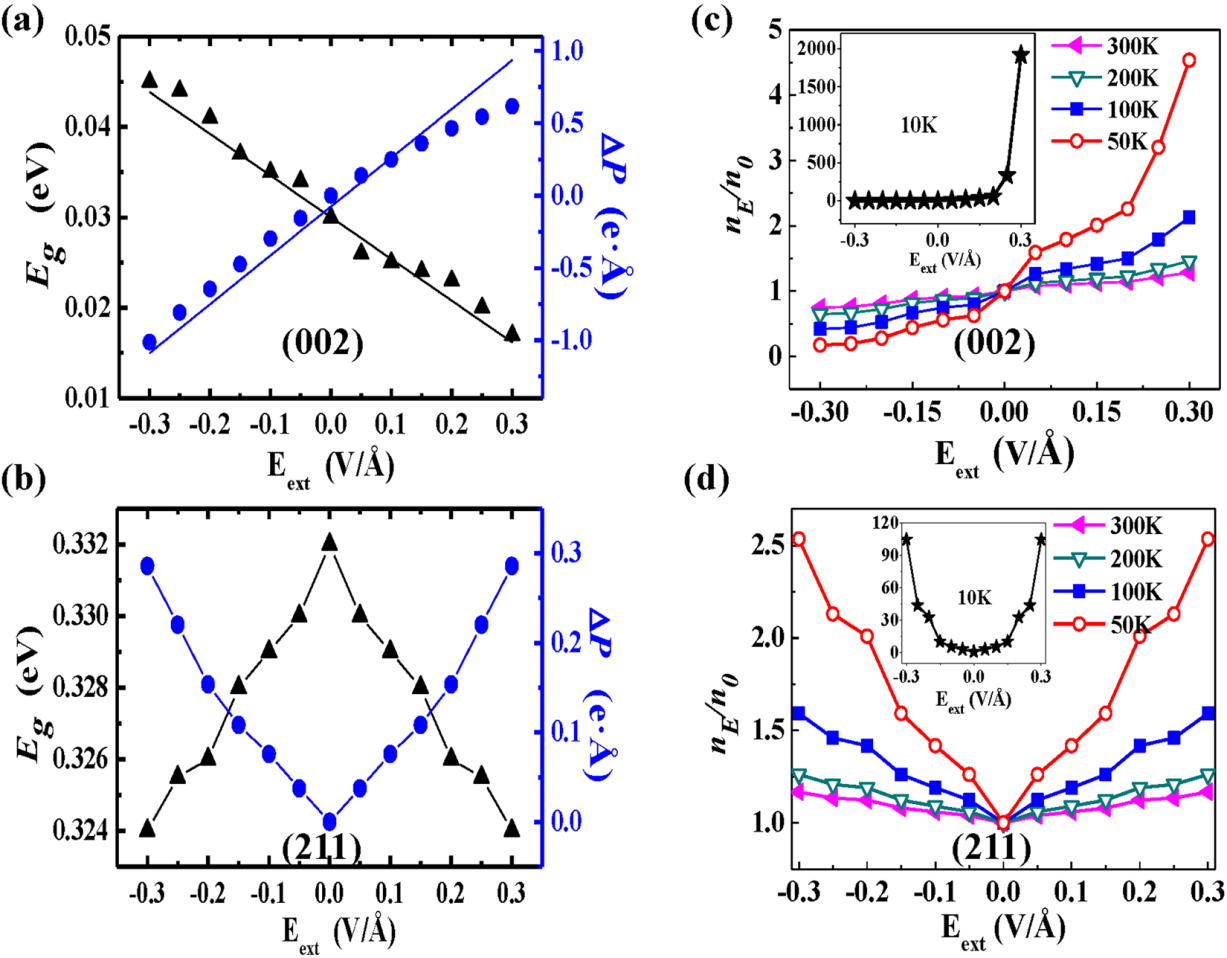
The band gap *E*_*g*_, the dipole shift (Δ*P*) and the intrinsic charge carrier concentration ratio *n*_*E*_/*n*_0_, and the resistivity *ρ* as a function of the external electric field *E*_*ext*_ for PbPdO_2_: (**a**) and (**c**) for (002) orientation; (**b**) and (**d**) for (211) orientation, respectively.

Figure 3(a,b) show the dependence of band gap *E*_*g*_ and the dipole shift Δ*P* (Electric-dipole moment changing defined as dipole shift Δ*P* is an alternative to charge transfer for quantitatively describing the charge rearrangement) on external electric field *E*_*ext*_ for PbPdO2 with (002) and (211) orientations, respectively. Here the external electric fields are all perpendicular to the stacking layers. As shown in Fig. 3(a), band gap of (002) orientation slab shows a monotonous decrease with increasing electric field from *E*_*ext*_ = −0.3 V/Å to *E*_*ext*_ = 0.3 V/Å. However, in contrast, (211) orientation slab has quite different band gap as a function of electric field. The band gap of (211) orientation slab decreases with increasing value of |*E*_*ext*_|, which displays a symmetrical electric field dependence of band gap, as shown in Fig. 3(b). For the intrinsic semiconductor, the charge charier concentration can be estimated using the Eq. (1) as follows^19^,

1 $$n\propto {T}^{3/2}{e}^{-\frac{{E}_{g}}{2{K}_{B}T}}$$

where *T* is temperature, *K*_B_ is Boltzmann constant. At *E*_*ext*_ = 0, let *n* = *n*_0_; *E*_*ext*_ ≠ 0, let *n* = *n*_E_. Figure 3(c,d) show the intrinsic charge carrier concentration ratio *n*_*E*_/*n*_0_ as a function of the external electric field *E*_*ext*_ for PbPdO_2_ with (002) and (211) orientations at *T* = 10 K, 50 K, 100 K, 200 K and 300 K, respectively. Here, the band gap data are taken from Fig. 3(a,b). From Fig. 3(c,d), it is found that the external electric field has great modulation on the charge carrier concentration and resistivity *ρ* $(\rho \propto \frac{1}{n})$, especially for low temperature and (002) orientation with small band gap and sensitive electric field modulation. For example, as *T* = 10 K, *E*_*ext*_ = 0.3 V/*Å*, the band gap reaches minimum value (0.015 eV) and the value of *n*_*E*_/*n*_0_ could be calculated up to 2000. Since resistivity (*ρ*) is in inverse proportion to carrier concentration (n), the resistivity is correspondingly decreases (1/2000) at *E*_*ext*_ = 0.3 V/Å when T = 10 K, which means that the electric field modulation of the resistivity might reach up to 3–4 orders of magnitude. The calculated results could well explain the origin of the observed colossal electroresistance in Wang’s work^6^. Therefore, it is suggested that the appearing of the colossal electroresistance of PbPdO_2_–based semiconductors may closely be associated with preferred orientation films with small band gap and sensitive electric field modulationof band gap.

**Figure 4:**
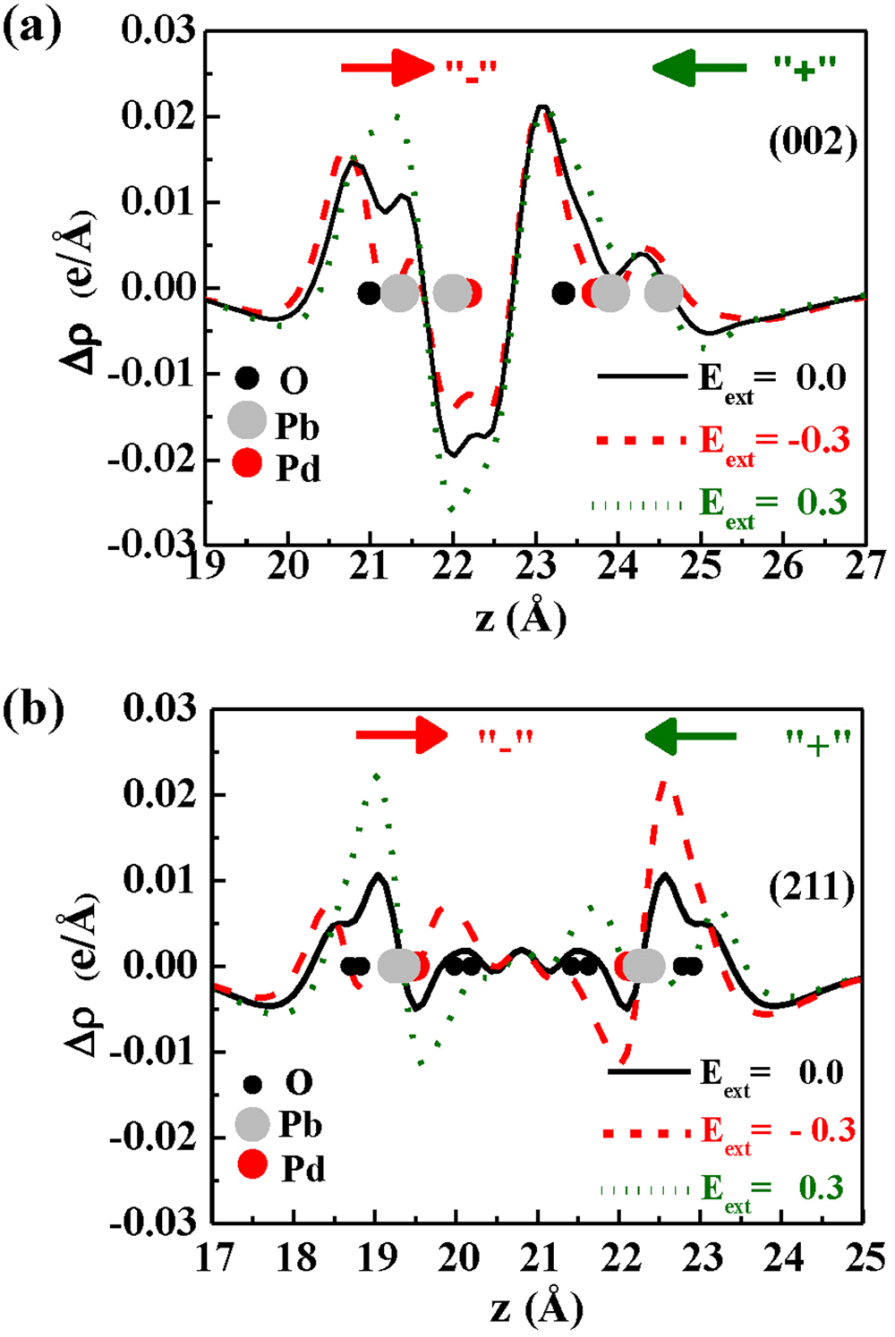
The plane-averaged electron density difference along the direction perpendicular to the slab with *E*_*ext*_ = 0.0, ±0.3 V/Å for (**a**) (002) and (**b**) (211) orientation slabs, respectively.

The re-distribution of charge in slabs is proposed to explain the potential mechanism of band gap modulation under electric filed. The charge density difference is usually to elucidate charge transition information, bonding structure and bonding strength during the bonding process of atom in slabs. Figure 4(a,b) present the charge density difference of (002) and (211) orientation slabs when the electric field is set as +0.3 V/Å, 0.0 and −0.3 V/Å, respectively. Here, the positive (“+”) direction in (002) orientation slab is against c-axis direction and the positive (“+”) direction in (211) orientation slab is against b-axis direction. It is known that the average charge density of surface charge difference of slabs along the external electric filed could be expressed as follows^20^,

2 $${\rm{\Delta }}\rho (z)={\rho }_{PbPd{O}_{2}}(z)-{\rho }_{Pb}(z)-{\rho }_{Pd}(z)-{\rho }_{O}(z)$$

where ${\rho }_{PbPd{O}_{2}}(z)$ is the average charge density at z in vertical PbPdO_2_ slab; *ρ*_*Pb*_(*z*), *ρ*_*Pd*_(*z*) and *ρ*_*O*_(*z*) are the average charge density of Pb, Pd and O atom at z, respectively. In the case of Pd-O bonding, when *E*_*ext*_ = 0, the electron cloud is mainly distributing around O atom due to polar covalent bonding of Pd-O. Because of the quantum-confined Stark effect^21–23^, the electrons would transfer from one side to the other side of atomic layers against the direction of the electric field, leading an overall net shifting in energy. Due to the asymmetry of Pd-O bonding, a spontaneous electric polarization exists along c-axis direction in (002) orientation slab. Thus, the equivalent field in slab is enhanced as the electric field is along c-axis direction, while it is weakened as the electric field is against c-axis direction, which results in the band gap increases or decreases due to Stark effect^21^. For (211) orientation slab, there does not exist spontaneous electric polarization along b-axis direction because of the inversion symmetry. A potential difference (*U* = −*d E* * *e*) between the two layer of slab is induced by the external electric field^15^, where E* is the total electric field (applied electric field plus inner electric field induced from redistribution of the charge), *d* represents the distance between neighboring layers, and *e* does the electron charge. As a result, the energy bands belonging to different atom layers will be separated from each other. The larger the applied electric field is, the larger the splitting value of the band is, and the narrower the band gap of the film is.

## Discussion

**Figure 5:**
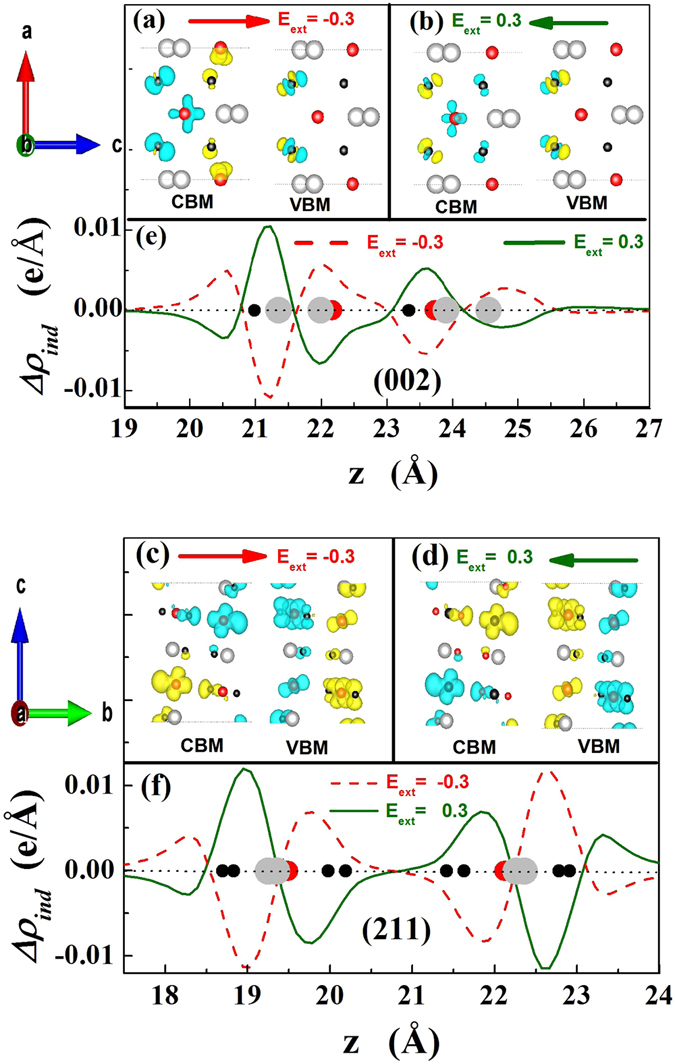
Side view of induced charge density of the CBM and VBM under external electric fields with isosurface value of 0.0009 e/au^3^ for (002) slab with (**a**) *E*_*ext*_ = −0.3 V/Å and (**b**) *E*_*ext*_ = 0.3 V/Å; for (211) slab with (**c**) *E*_*ext*_ = −0.3 V/Å and (**d**) *E*_*ext*_ = 0.3 V/Å. The yellow and blue colors indicate electron accumulation and depletion, respectively. The induced plane-averaged charge density change, ${\rm{\Delta }}{\rho }_{ind}(z,{E}_{ext})={\rho }_{PbPd{O}_{2}}(z,{E}_{ext})-{\rho }_{PbPd{O}_{2}}(z,0)$, with *E*_*ext*_ = ±0.3 V/Å for (**e**) (002) slab; (**f**) (211) slab.

It was reported that the external electric field would induce the charge transfer in slab and the electrostatic potential felt by electrons correspondingly changes, resulting in the energy-level shifting of CBM and VBM^21, 23, 24^. Figure 5 shows charge density difference of VBM and CBM and field-induced plane-averaged charge density (Δ*ρ*_*ind*_) caused by charge redistribution of (002) and (211) orientation slabs under the external electric field. It is suggested that field-induced plane-averaged charge density (Δ*ρ*_*ind*_) would be responsible for the band gap variations, which induces additional energy barrier due to the electron transfer of CBM and VBM^25, 26^. The dipole change (Δ*P*) in slab along the external field could be calculated as following equations^27, 28^,

3 $${\rm{\Delta }}P=-{\int }_{z\in unitcell}{\rm{\Delta }}{\rho }_{ind}({\rm{z}})\cdot z\cdot dz$$

4 $${\rm{\Delta }}{\rho }_{ind}(z)={\rho }_{ext}(z)-{\rho }_{0}(z)$$

It has been reported that the change of dipole energy barrier (Δ*D*) of the system is proportional to the dipole-moments change (Δ*P*), namely Δ*D* ∝ −Δ*P*, which results in the energy-level shifting of CBM and VBM^25, 26^. As shown in Fig. 3(a,b), the surface dipole shift Δ*P* as a function of the external electric field (*E*_*ext*_) is just inversely proportional to the change of band gap, which means that the band gap modulation results mainly from the change of Δ*P* (Δ*D*) induced by the charge transfer. Moreover, the electron accumulation and depletion could clearly be observed in Fig. 5(a–d). As shown in Fig. 5(a), as the electric field direction is along with c-axis direction of (002) orientation slab, the effective charge polarization in conduction band is against to electric field, which enhances electron energy in the conduction band and consequently the bottom of the conduction band shifts to higher-energy direction. The effective charge polarization in the valence band is along to electric field, which decreases electron energy in the valence band and consequently the top of the valence band shifts to lower-energy direction. As a result, the band-gap of (002) orientation slab is increased. Similarly, as seen in Fig. 5(c), when the electric field direction is along with b-axis direction of (211) orientation slab, the effective charge polarization in the valence band is against to electric field, which increases electron energy in the valence band and consequently the top of the valence band shifts to higher-energy direction. However, the effective charge polarization in the conduction band is perpendicular to the electric field, which could not lead to the shift of the bottom of the conduction band. Thus, the electric field modulation of band-gap for (211) orientation slab becomes less.

## Conclusions

In summary, the electric field modulations of band-gap for (002) and (211) orientation slabs in PbPdO_2_ are investigated by first-principles calculations. A different band-gap as a function of the external electric field is found in (002) and (211) orientation slabs. The band gap of (002) orientation slab shows a monotonous decrease with increasing electric field when the electric field is along c-axis direction of (002) orientation slab, while the band gap increases with increasing electric field as the electric field is against c-axis direction. The band gap of (211) orientation slab displays a monotonous decrease with increasing absolute electric field. Moreover, the electric field modulation of the resistivity up to 3–4 orders of magnitude is found, which reveals the origin of the colossal electroresistance. Therefore, it is suggested that the appearing of the colossal electroresistance of PbPdO_2_–based semiconductors may closely be associated with preferred orientation films with small band gap and sensitive electric field modulation of band gap. At last, the band-gap modulations under electric field could be well explained by charge polarization theory, which might be significant for preparing PbPdO_2_-based half semimetals.

## Methods

In this work, the first-principle calculations were carried out by using Vienna *ab initio* simulation package (VASP), and projector augmented wave method (named as PAW) was used in the electron-ion interactions^29, 30^. The exchange-correlation energy was considered according to Perdew-Burke-Ernzerhof (PBE) formulation of generalized gradient approximation (GGA)^31^. The cut-off energy was chosen to be 550 eV. Optimizations of the structures were implemented by relaxing the positions of all the atoms in the cell until the convergence tolerance of force on each atom is less than 0.001 eV/Å with the Gaussians smearing method. In order to simulate the two-dimensional infinite sheet, periodic boundary condition was used. 23 × 15 × 1 and 23 × 1 × 17 Monkhorst-Pack’s meshes were used in calculation of density of states (DOS) for the (002) and (211) orientation slabs, respectively. The slab (plate crystal) with around 35 Å vacuum layer was adopted.
